# Circadian disruption in lung fibroblasts enhances NF‐κB activity to exacerbate neutrophil recruitment

**DOI:** 10.1096/fj.202201456R

**Published:** 2023-01-09

**Authors:** Shannon L. Cox, James R. O'Siorain, Yan He, Ronan Lordan, Amruta Naik, Soon Yew Tang, Shaon Sengupta, Garret A. FitzGerald, Richard G. Carroll, Annie M. Curtis

**Affiliations:** ^1^ Curtis Clock Laboratory, School of Pharmacy and Biomolecular Sciences (PBS) Royal College of Surgeons in Ireland (RCSI) Dublin Ireland; ^2^ Institute of Functional Nano and Soft Materials (FUNSOM) Jiangsu Key Laboratory for Carbon‐Based Functional Materials and Devices Soochow University Suzhou China; ^3^ Institute of Translational Medicine and Therapeutics (ITMAT) University of Pennsylvania Philadelphia Pennsylvania USA; ^4^ Children's Hospital of Pediatrics Philadelphia Pennsylvania USA; ^5^ Department of Paediatrics University of Pennsylvania Perelman School of Medicine Philadelphia Pennsylvania USA; ^6^ Tissue Engineering Research Group (TERG) Royal College of Surgeons in Ireland (RCSI) Dublin Ireland

**Keywords:** *Bmal1^−/−^*, circadian rhythms, fibroblasts, immune signaling, inflammation, lung

## Abstract

Fibroblasts are stromal cells abundant throughout tissues, including the lungs. Fibroblasts are integral coordinators of immune cell recruitment through chemokine secretion. Circadian rhythms direct the recruitment of immune cells to the lung, which in turn impacts response to infection and survival. Although fibroblasts display robust circadian rhythms, the contribution of the fibroblast molecular clock to lung‐specific migration of immune cells and recruitment remains to be established. Mice challenged intranasally with lipopolysaccharide (LPS) at dusk showed increased expression of the pro‐inflammatory cytokine IL‐1β and chemokine CXCL5 in the lung, which was accompanied by increased neutrophil recruitment. Primary lung fibroblasts with knockdown of the core clock gene *Bmal1* and immortalized *Bmal1* ^−/−^ lung fibroblasts also displayed increased *Cxcl5* expression under IL‐1β stimulation. Conditioned media obtained from IL‐1β‐stimulated *Bmal1* ^−/−^ immortalized fibroblasts‐induced greater neutrophil migration compared with *Bmal1* ^+/+^ lung fibroblast controls. Phosphorylation of the NF‐κB subunit, p65, was enhanced in IL‐1β‐stimulated *Bmal1* ^−/−^ lung fibroblasts, and pharmacological inhibition of NF‐κB attenuated the enhanced CXCL5 production and neutrophil recruitment observed in these cells. Collectively, these results demonstrate that *Bmal1* represses NF‐κB activity in lung fibroblasts to control chemokine expression and immune cell recruitment during an inflammatory response.

**Abbreviations**
BAL: bronchoalveolar lavage
CM: conditioned media
COPD: chronic obstructive pulmonary disorder
CSF1/2/3: colony‐stimulating factor 1/2/3
CT: circadian time
ELISA: enzyme‐linked immunosorbent assay
IPF: idiopathic pulmonary fibrosis
LPS: lipopolysaccharide
PS: post‐shock
qPCR: quantitative PCR
VEGF: vascular endothelial growth factor
ZT: zeitgeber time

## 1 INTRODUCTION

Circadian rhythms are daily oscillations in biological processes, which peak and trough once every 24‐h.^1^ Within each cell, circadian rhythms are controlled by a system of positive and negative transcriptional‐translational feedback loops known as the endogenous “molecular clock” comprised of the core clock components.^2^ The bHLH‐PAS transcription factor *Bmal1*, a key component of the positive arm of the molecular clock, is central to this time‐keeping system as its deletion leads to disruption of the molecular clock and associated outputs.^3^ The molecular clock aligns cellular processes with daily environmental cues, such as light and eating.^4^ Shift‐work and erratic sleeping/eating patterns, both of which are prevalent in modern society, leads to circadian misalignment and disruption and is associated with a host of chronic inflammatory diseases.^5^, ^6^, ^7^, ^8^

The lung is an important immunological organ that displays significant circadian rhythmicity, while numerous respiratory pathologies have time‐of‐day variation in disease severity.^9^, ^10^ Asthma and chronic obstructive pulmonary disorder (COPD) symptoms worsen during the early morning in humans,^11^, ^12^, ^13^ while smoking and shift‐work dysregulate rhythms in the human lung, increasing lung inflammation and fibrosis.^6^, ^14^ Mice infected with influenza have varied survival depending on the timing of infection.^15^ Fibroblasts are the most abundant cell type within the lung parenchyma and recruit macrophages and neutrophils to propagate the inflammatory response.^16^, ^17^, ^18^ Dysregulation of lung fibroblasts leads to increased inflammation and progression of lung pathologies including asthma,^19^ COPD,^20^ idiopathic pulmonary fibrosis (IPF),^6^ and cancer,^21^ conditions which are also associated with circadian disruption.^6^, ^8^, ^11^, ^12^, ^13^ Current treatments for many of these fibroblast‐related pathologies focus on mitigating symptoms as opposed to curing the disease, indicating an urgent need to understand the molecular components underpinning these pathologies.

Many chemokines produced during the pulmonary inflammatory response influence immune cell trafficking to the lung. One such chemokine is CXCL5, the levels of which display robust circadian variation, attracts neutrophils to the lung in a time‐of‐day manner.^22^ CXCL5‐induced neutrophil recruitment has been associated with lung cancer progression.^23^ Increased CXCL5 levels correlate with decreased lung function in COPD patients and levels increase further with smoking.^24^, ^25^ Transcription of the *Cxcl5* gene is mediated through the transcription factor NF‐κB.^26^ Gene transcription can occur through numerous stimuli, including the pro‐inflammatory cytokine IL‐1β, produced predominantly by macrophages.^27^ Interestingly, IL‐1β production in macrophages is under circadian control^28^, ^29^ and levels are heightened in respiratory infections,^30^ asthma,^31^ and COPD.^32^

Herein, we describe a novel pathway whereby IL‐1β induced CXCL5 production in lung fibroblasts is NF‐κB dependent and leads to neutrophil recruitment. We show that *Bmal1* in lung fibroblasts supresses NF‐κB activity, which limits CXCL5 production and neutrophil recruitment in a time‐of‐day‐dependent manner. This study highlights the importance of the fibroblast molecular clock in terms of immune cell regulation in the lung under inflammatory conditions.

## 2 MATERIALS AND METHODS

### 2.1 Transgenic mice

Inducible *Bmal1* ^*KD*^ mice (*Bmal1* ^*fl*/*fl*^ *ERcre* ^+^) were generated by crossing *Bmal1* ^*fl*/*fl*^ mice with *ERCre* ^+^ mice. Knockdown of *Bmal1* was achieved via intraperitoneal tamoxifen injection (100 mg/kg, T5648, Merck) for 5 consecutive days. Stock solution of tamoxifen (1 g) was reconstituted with 15% ethanol and 85% peanut oil to give a 100 mg/ml solution that was further diluted to 50 mg/ml prior to administration. Mice were injected at 8 weeks old and left for at least 2 weeks prior to use in experiments. Mice negative for *Cre* (*Bmal1* ^*fl*/*fl*^ *ERcre* ^−^; *Bmal1* ^*WT*^) were used as controls. Mice were housed in CMU in Trinity College Dublin. All mice were maintained according to European Union regulations and the Irish Health Products Regulatory Authority. Experiments were performed under Health Products Regulatory Authority license with approval from the Trinity College Dublin BioResources Ethics Committee. All animal procedures were in line with the EU Directive 2010/63/EU and with a project authorization number AE19136/P007. All animal studies were approved by the University of Pennsylvania Institutional Animal Care and Use Committee and met the stipulations of the Guide for the Care and Use of Laboratory Animals.

### 2.2 Intranasal LPS treatment

Age‐matched wild type C57BL/6J mice were housed in circadian cabinets set in opposing 12:12 light/dark cycles with food and water available ad libitum. After acclimatization, mice were subjected to constant darkness for 1–2 days in the cabinets prior to intranasal LPS administration to mitigate any masking effects due to light. For intranasal LPS administration, mice were first gently anesthetized with 2.5% isoflurane. LPS (3 mg/kg, from Escherichia coli O111:B4, L2639, Merck) or PBS was then pipetted intranasally in a volume of 40 μl at either circadian time 0 (CT0; subjective lights on, start of rest phase in mice) or CT12 (subjective lights off, start of active phase in mice) for 6 or 24 h. After this time, mice were euthanized and bronchoalveolar lavage (BAL) and lungs were harvested.

### 2.3 Bronchoalveolar lavage, lung digestion, fibroblast isolation

BAL was obtained by installing a cannula into the trachea and then administering 1 ml EDTA (50 mM) and protease inhibitor (1 in 100; Halt™ Protease Inhibitor Cocktail, EDTA‐free (100X), 78425, Thermo Fisher Scientific) in PBS. The heart was then perfused with 10 ml ice‐cold PBS and the lungs were either taken for qPCR analysis or inflated with 10 ml dissociation media (DMEM, D5796, Merck; 2‐mercaptoethanol, M3148, Merck; pen/strep, P4333, Merck; DNase I, 1 mg/ml, 11284932001, Merck; liberase, 5 mg/ml 5401119001, Merck) and dissected from the mice. The dissociated lung tissue was then passed through a cell strainer, centrifuged, and red blood cells were lysed using RBC lysis buffer (R7757, Merck). The BAL was then centrifuged at 1500 rpm for 5 min before the supernatants were removed for ELISA analysis of IL‐1β (DY401, Bio‐Techne) and CXCL5 (DY443, Bio‐Techne), and the cell pellet was combined with lung cell suspensions. A single cell suspension was obtained for analysis via flow cytometry.

### 2.4 Bone marrow cell isolation

Bone marrow‐derived cells were isolated from the legs of male or female C57BL/6J mice. Mice were euthanized via carbon dioxide inhalation followed by cervical dislocation. Legs were removed and once clean, femurs and tibias were flushed using HSW FINE‐JECT hypodermic needles (HSWNH2558‐100EA, Merck) with media (DMEM, 10% FBS, 1% pen/strep) warmed to 37°C. Cells were collected in 50 ml falcon tubes and centrifuged at 1500 rpm for 5 min at room temperature. The supernatant was discarded, and the cell pellet was re‐suspended in 2 ml Red Blood Cell Lysing Buffer Hybri‐Max™ (R7757‐100ML, Merck) for 5 min. After 5 min, 20 ml of media was added to the samples, and the cell suspension was pipetted through a 40 μm cell strainer into a new 50 ml falcon tube and centrifuged at 1500 rpm for 5 min at room temperature. The single cell suspension obtained was then used for the transwell migration assay.

### 2.5 Cell culture

Lung derived‐*Bmal1* ^+/+^, *Bmal1* ^−/−^, *Cry1* ^+/+^ *Cry2* ^+/+^, and *Cry1* ^−/−^ *Cry2* ^−/−^ primary murine fibroblasts homozygous for PER2::LUCIFERASE were used in this study. These fibroblasts were obtained from 6‐month‐old mice and immortalized by serial passage (donated by the John O'Neill Lab in Cambridge), referred to in this study as immortalized lung fibroblasts.^33^, ^34^

For primary lung fibroblasts, lungs were obtained as outlined above (see lung digestion) and once a single‐cell suspension was achieved, cells were left to attach in T75 flasks in DMEM. On day 4, media was replaced with fresh DMEM, and by day 8 fibroblasts were obtained and passaged into T175 flasks. Cells were then trypsinized and plated in 12‐well plates at a concentration of 0.5 × 10^5^ cells/ml for experiments.

Both immortalized and primary lung fibroblasts were stimulated with IL‐1β (10 ng/ml, rm IL‐1β/rm IL1F2, 12 340 015, ImmunoTools) for indicated times.

### 2.6 Western blotting and chemokine array

For Western blotting, cells were lysed in 1 × Laemmli buffer containing DTT (1:20, 43816, Merck) and benzonase nuclease (1:10 000, sc‐202391, Santa Cruz) and placed in a heating block for 5 min: 10 μl sample was resolved on 12% gel and transferred onto nitrocellulose membranes (GE10600004, Merck). Membranes were blocked using 5% milk for 1 h and incubated with the following primary antibodies overnight: Phospho‐NF‐κB p65 (Ser536) Antibody (1:500, 3031S, Cell Signaling Technology); IκBα (L35A5) Mouse mAb (Amino‐terminal Antigen) (1:1000, 4814S, Cell Signaling Technology); BMAL1 (D2L7G) Rabbit mAb (1:1000, 14020S, Cell Signaling Technology); β‐Actin (8H10D10) mouse mAb (1:10 000, 3700S, Cell Signaling Technology). Membranes were incubated for 2 h with relevant secondary antibodies: Peroxidase AffiniPure Goat Anti‐Rabbit IgG (1:2000, 111‐035‐144, Jackson ImmunoResearch) and Peroxidase AffiniPure Goat Anti‐Mouse IgG (1:2000, 115‐035‐146, Jackson ImmunoResearch).

For the Proteome profiler mouse XL chemokine array kit (ARY028, Bio‐Techne), supernatants were generated by stimulating immortalized *Bmal1* ^+/+^ and *Bmal1* ^−/−^ lung fibroblasts with IL‐1β or vehicle control for 24‐h. Supernatants were removed and the Proteome profiler mouse XL chemokine array kit was used according to manufacturer's instructions.

Proteins for Western blotting and chemokine array kit were detected via chemiluminescence using Amersham 680 Imager (GE Healthcare). Densitometry was carried out using ImageLab software (V.6.1, BioRad).

### 2.7 Transwell migration assay and imaging

Immortalized *Bmal1* ^+/+^ and *Bmal1* ^−/−^ lung fibroblasts were plated at a concentration of 0.5 × 10^5^ cells/ml in 12‐well plates in Opti‐MEM™ I Reduced Serum Medium (31985047, Thermo Fisher Scientific). Fibroblasts were left untreated or treated with the NF‐κB inhibitor JSH‐23 (10 μM, J4455‐5MG, Merck) for 1 h prior to stimulation with IL‐1β (10 ng/ml) or vehicle control for 24‐h. After 24‐h of IL‐1β stimulation, supernatants (1 ml) were removed, centrifuged at 1500 rpm for 10 min (to remove cell debris), and 900 μl was pipetted into a 24‐well Carrier Plate with Cell Culture Inserts (141004, Thermo Fisher Scientific). Bone marrow‐derived cells were isolated from the legs of male or female C57BL/6J mice and placed into hanging well inserts into the wells containing fibroblast supernatants and left to migrate overnight. Cells were then either stained with Calcein AM (2 μM, C3099, Thermo Fisher Scientific) according to manufacturer's instructions and imaged using a Nikon Eclipse TS100 fluorescent microscope or stained for flow cytometry analysis. For cell quantification, images were analyzed using FIJI Software (ImageJ).

### 2.8 RNA isolation and qPCR

Total RNA was isolated (Purelink RNA mini‐isolation kit 250, 12183025, Thermo Fisher Scientific) and reverse transcribed (High‐Capacity cDNA Reverse Transcription Kit, 4368813, Thermo Fisher Scientific) using ProFlex system (ProFlex™ 3 × 32‐well PCR System). For whole lung samples from inducible *Bmal1* ^*KD*^ mice (*Bmal1* ^*fl*/*fl*^ *ERcre* ^+^) and corresponding *Bmal1* ^*WT*^ mice (*Bmal1* ^*fl*/*fl*^ *ERcre* ^−^), lungs were harvested and stored in Invitrogen™ RNAlater™ Stabilization Solution (AM7020, Thermo Fisher Scientific) according to manufacturer's protocol. For RNA isolation from whole lung samples, the tissues were first homogenized into a single cell suspension following the protocol outlined in PureLink™ RNA Mini Kit. RNA was subsequently isolated from lung single cell suspension, or plated cells following the protocol outlined in PureLink™ RNA Mini Kit. For qPCR, the following primers were used: *18s* forward, 5′‐CCCTCTATGGGCTCGAATTT‐3′, reverse, 5′‐GGATGTGAAGGATGGGAAGT‐3′; *Cxcl5* forward, 5′‐TGCCCTACGGTGGAAGTCAT‐3′, reverse, 5′‐AGCTTTCTTTTTGTCACTGCCC‐3′; *Il1b* forward, 5′‐TGGCAACTGTTCCTG‐3′, reverse, 5′‐GGAAGCAGCCCTTCATCTTT‐3′; *Bmal1* forward, 5′‐TGCAATGTCCAGGAAGTTAGAT‐3′, reverse, 5′‐GTTTGCTTCTGTGTATGGGTTG‐3′; *Clock* forward, 5′‐CCTATCCTACCTTGGCCACACA‐3′, reverse, 5′‐TCCCGTGGAGCAACCTAGAT‐3′; *Nr1d1* forward, 5′‐GAGAGGCCATCACAACCTCC‐3′, reverse, 5′‐ACACCACCTGTGTTGTTATTGG‐3′; *Nr1d2* forward, 5′‐CGGATCACATGGTCGAGGAG‐3′, reverse, 5′‐GCAATCACACCTCCTGCGTT‐3′; *Per1* forward, 5′‐GAGCTTCTGGGTTGCGGG‐3′, reverse, 5′‐TTAATCACGACACCTGGCCG‐3′; *Per2* forward, 5′‐ATGCTCGCCATCCACAAGA‐3′, reverse, 5′‐GCGGAATCGAATGGGAGAAT‐3′; *Cry1* forward, 5′‐CTCGGTAGAGGAAGTCGGGG‐3′, reverse, 5′‐TGAAGCAAAAATCGCCACCT‐3′; *Cry2* forward, 5′‐GCAAGGACTCCTGAGACTGGA‐3′, reverse, 5′‐CGTCTGTTGGTGATTGGCTT‐3′ (all for use with PowerUp™ SYBR® Green Master Mix, A25778, Thermo Fisher Scientific). RT qPCR reaction was run using Applied Biosystems 7900HT PCR system.

### 2.9 siRNA knockdown of *Bmal1*

Immortalized *Bmal1* ^+/+^ lung fibroblasts were plated at a density of 0.5 × 10^4^ cells/ml in a 24‐well plate. Fibroblasts were then treated with 50, 100, or 200 nM *Arntl* Silencer Select siRNA (5 nmol, 4390771, Ambion) in Lipofectamine RNAiMAX Transfection Reagent (13778150, Thermo Fisher Scientific) and Opti‐MEM™ I Reduced Serum Medium (31985047, Thermo Fisher Scientific) for 24 or 48 h. Samples were subsequently lysed and analyzed for protein and mRNA expression via western blotting and qPCR respectively.

### 2.10 Flow cytometry

For whole lung samples and bone marrow cells, samples were centrifuged and resuspended in PBS. Cells were incubated with Fc block (1:500, Purified anti‐mouse CD16/32 Antibody, clone 93, 101301, BioLegend), Zombie NIR Fixable Viability Kit (1:500, 423105, BioLegend), or Zombie Aqua Fixable Viability Kit (1:500, 423101, BioLegend), and indicated antibodies at room temperature for 20 min: FITC anti‐mouse Ly‐6G Antibody (1:50, clone 1A8, 127605, BioLegend); PE anti‐mouse CD170 (Siglec‐F) Antibody (1:50, clone S17007L, 155505, BioLegend); PerCP/Cyanine5.5 anti‐mouse Ly‐6C Antibody (1:50, clone HK1.4, 128011, BioLegend); PE/Cyanine7 anti‐mouse CD45 Antibody (1:50, clone S18009F, 157205, BioLegend); APC anti‐mouse CD11c Antibody (1:50, clone N418, 117309, BioLegend); Alexa Fluor® 700 anti‐mouse/human CD11b Antibody (1:50, clone M1/70, 101222, BioLegend); Brilliant Violet 421™ anti‐mouse/human CD11b Antibody (1:50, clone M1/70, 101235, BioLegend). Cells were then washed with PBS containing 2% FBS and 1 mM EDTA (flow buffer) and fixed using Cyto‐Fast™ Fix/Perm Buffer Set (426803, BioLegend) according to manufacturer's protocol. Cells were then washed with flow buffer, centrifuged at 300 *g* for 5 min, resuspended in 200 μl flow buffer, and left at 4°C overnight. Neutrophils were defined as CD45+SiglecF‐CD11c‐Ly6G+CD11b+; Alveolar macrophages were defined as CD45+SiglecF+CD11c+; Lung‐derived inflammatory monocytes were defined as CD45+SiglecF‐CD11b+Ly6C+; Bone marrow‐derived macrophages/monocytes were defined as CD11b+Ly6G‐Ly6C+. Data were obtained using FACS Canto II and analyzed using FlowJo (version 8).

### 2.11 Serum shock

To synchronize immortalized lung *Bmal1* ^+/+^ fibroblasts in vitro, cells were first plated in DMEM (10% FBS, 1% pen/strep). Media was then replaced with 50% DMEM and 50% horse serum for 2 h. Media was then changed back to normal DMEM (10% FBS, 1% pen/strep). Fibroblasts were then left unstimulated or stimulated with IL‐1β (10 ng/ml) at 12‐h post‐shock (equivalent Zeitgeber Time 0; ZT0) or 24‐h post‐shock (equivalent ZT12). After this time, cells were lysed for qPCR analysis and supernatants taken for ELISA analysis.

### 2.12 Lumicycle

PER2::Luciferase immortalized *Bmal1* ^+/+^ lung fibroblasts were plated at a density of 1.5 × 10^5^ cells/ml in 35 mm dishes in DMEM (10% FBS, 1% Pen/Strep). The following day, *Bmal1* ^+/+^ lung fibroblasts were synchronized by serum shock as described above. To monitor circadian rhythmicity, synchronization media was replaced with Lumicycle recording media (DMEM, l‐glutamine, glucose (1000 mg), without phenol red and sodium bicarbonate (D‐2902, Merck), 10% FBS, 1% Pen/Strep, beetle luciferin potassium salt (0.1 mM, Promega E1603)). The 35 mm dishes were sealed using 40 mm coverslips with Dow Corning® high‐vacuum silicone grease. Bioluminescence was recorded with the 32‐channel Lumicycler by Actimetrics for 5 days beginning at 12 h post serum shock. Analysis was performed using the Actimetrics Lumicycle Analysis program.

### 2.13 Promoter analysis

The R package “RCISTarget” was used to identify transcription factor binding motifs enriched within 500 base pairs of the transcription start site of *Cxcl5*.^35^ (https://rdrr.io/github/aertslab/RcisTarget/man/motifAnnotations.html) (https://resources.aertslab.org/cistarget).

### 2.14 Statistical analyses

GraphPad Prism 8 (GraphPad Software) was used for statistical analysis. Data were analyzed using Kruskal–Wallis with Dunn's multiple comparisons, one‐way ANOVA with Tukey's multiple comparisons, or unpaired *t* test Differences were considered statistically significant when *p* < .05 (*), *p* < .01 (**), *p* < .001 (***), *p* < .0001 (****). Data shown represent mean values per experimental group ± SEM.

## 3 RESULTS

### 3.1 Mice administered intranasal LPS at CT12 have increased *Cxcl5* mRNA expression, IL‐1β production, and enhanced neutrophil recruitment to lungs

**FIGURE 1:**
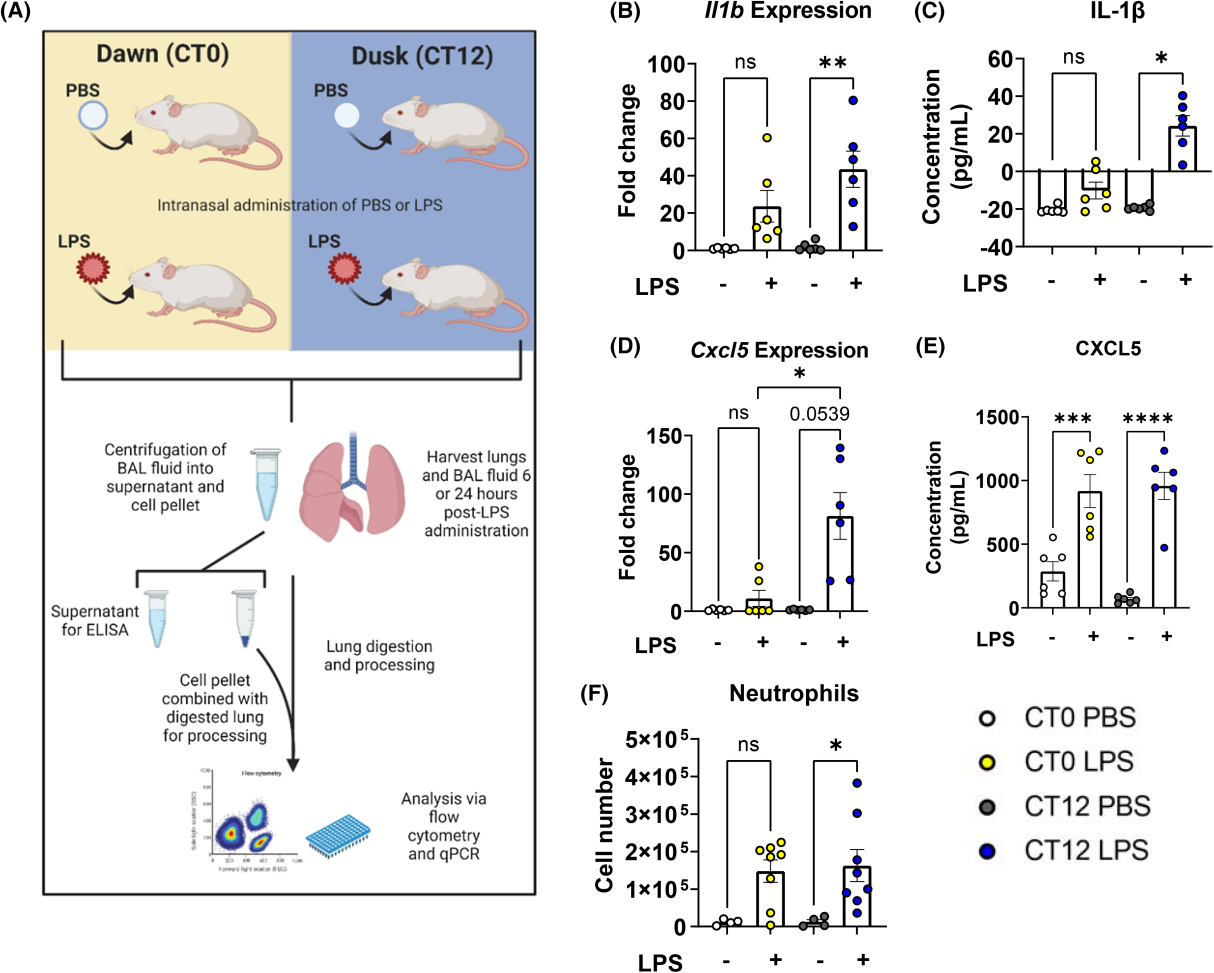
Mice administered intranasal LPS at CT12 have increased *Cxcl5* expression and enhanced neutrophil recruitment. (A) Schematic illustrating experimental design of in vivo experiment. (B) mRNA expression of *Il1b* and (D) *Cxcl5* in whole lung tissues harvested from C57BL/6J mice 6 h after PBS or LPS (3 mg/kg) administration at CT0 or CT12 (*n* = 6 per group). (C) Protein expression of IL‐1β and (E) CXCL5 in BAL samples collected from the same experiment. (F) Neutrophil numbers recruited to the lung 24 h after PBS (*n* = 4) or LPS (*n* = 8) administration at CT0 or CT12. (B–D) Statistical analyses were conducted using Kruskal–Wallis with Dunn's multiple comparisons test and (E, F) one‐way analysis of variance (ANOVA) with Tukey's multiple comparisons test. Data are expressed as mean values per experimental group ± SEM. *p* < .05 (*), *p* < .01 (**), *p* < .001 (***), *p* < .0001 (****). (A) Created with BioRender.com.

To examine the time‐of‐day effect of intranasal LPS administration on the immune response, male C56BL/6J mice were exposed to LPS at either CT0 (subjective lights on; start of rest phase), or CT12 (subjective lights off; start of active phase) (Figure 1A). We first examined *Bmal1* mRNA expression in the lungs of mice at zeitgeber time (ZT) 0 and ZT12, and found *Bmal1* expression to be significantly lower at ZT12 compared with ZT0 (Figure S1D), a result that has also been reported elsewhere.^36^ Mice administered LPS at CT12 for 6 h had significantly increased *Il1b* mRNA and protein expression in the lung, while there was no significant increase in *Il1b* mRNA and protein expression in mice administered LPS at CT0 (Figure 1B,C). Similarly, mice administered LPS for 6 h at CT12 had greater induction of *Cxcl5* mRNA compared with LPS administered at CT0 (Figure 1D). While the protein expression of CXCL5 was not significantly different between mice administered LPS at CT0 and CT12, CXCL5 induction at CT12 was more robust (Figure 1E). We next decided to examine innate immune cell recruitment to the lung including neutrophils, alveolar macrophages, and inflammatory monocytes in mice administered intranasal LPS either at CT0 or CT12 for 24 h (Figure S1A–C). Neutrophils were the only cell type to display a significant increase in recruitment to the lung following LPS at CT12, while no significant increase was observed with LPS at CT0 (Figure 1F). This was of interest given that the main function of CXCL5 is neutrophil recruitment and is consistent with the observed higher mRNA expression of this gene with 6 h of IL‐1β stimulation. Overall, we observed increased *Cxcl5* mRNA expression, IL‐1β protein expression, and neutrophil recruitment at CT12 when *Bmal1* expression is low.

### 3.2 Loss of *Bmal1* in lung fibroblasts leads to increased CXCL5 expression

**FIGURE 2:**
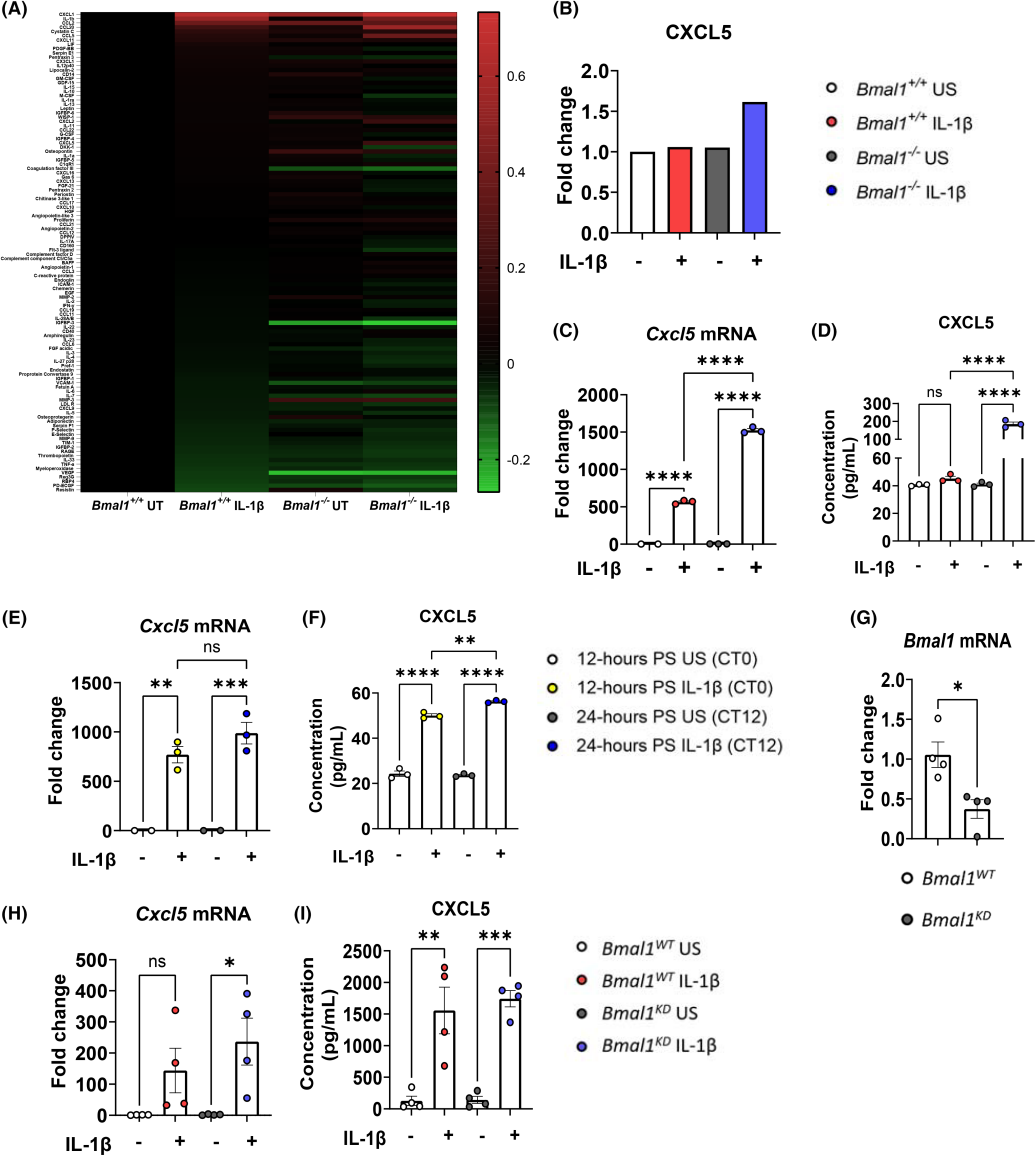
Loss of *Bmal1* in lung fibroblasts leads to increased CXCL5 expression. (A) Heatmap of cytokines and chemokines from immortalized *Bmal1* ^+/+^ and *Bmal1* ^−/−^ lung fibroblasts unstimulated or stimulated with IL‐1β (10 ng/ml) for 24 h (*n* = 1). (B) Densitometry of CXCL5 from chemokine array (A) (*n* = 1). (C) mRNA and (D) protein expression of CXCL5 in immortalized *Bmal1* ^+/+^ and *Bmal1* ^−/−^ lung fibroblasts unstimulated or stimulated with IL‐1β for 24 h (*n* = 3). (E) mRNA and (F) protein expression of CXCL5 in synchronized immortalized *Bmal1* ^+/+^ lung fibroblasts unstimulated or stimulated with IL‐1β at 12‐h post‐shock (equivalent CT0) or 24‐h post‐shock (equivalent CT12) for 6 h (*n* = 3). (G) mRNA expression of *Bmal1* in primary *Bmal1* ^*WT*^ and *Bmal1* ^*KD*^ lung fibroblasts (*n* = 4). (H) mRNA and (I) protein expression of CXCL5 in *Bmal1* ^*WT*^ and *Bmal1* ^*KD*^ primary lung fibroblasts unstimulated or stimulated with IL‐1β for 24 h (*n* = 4). (C–F, H, I) Statistical analyses were conducted using one‐way analysis of variance (ANOVA) with Tukey's multiple comparisons test, and (G) via unpaired *t* test. Data are expressed as mean values per experimental group ± SEM. *p* < .05 (*), *p* < .01 (**), *p* < .001 (***), *p* < .0001 (****).

To investigate the role of lung fibroblasts in the observed time‐of‐day immune response to LPS, we obtained immortalized *Bmal1* ^−/−^ and *Bmal1* ^+/+^ lung fibroblasts. We determined loss of *Bmal1* mRNA and protein in these cells and examined expression levels of the other core clock genes (Figure S2A,B). We performed a chemokine array to fully characterize the inflammatory signature of these cells when stimulated by IL‐1β (Figure 2A, Figure S2C; Data Table S1). Interestingly, CXCL5 showed up as one of the most highly expressed chemokines in *Bmal1* ^−/−^ lung fibroblasts in response to IL‐1β (Figure 2B). Both mRNA expression and protein levels of CXCL5 were significantly elevated in IL‐1β‐stimulated *Bmal1* ^−/−^ lung fibroblasts compared with IL‐1β‐stimulated *Bmal1* ^+/+^ lung fibroblasts (Figure 2C,D). Other chemokines that were elevated in IL‐1β‐stimulated *Bmal1* ^−/−^ lung fibroblasts compared with *Bmal1* ^+/+^ lung fibroblasts included CXCL1 and CXCL2, which are also prominent neutrophil recruiters (Figure S2D). Other inflammatory mediators such as CSF1, CSF2, CSF3, and VEGF were reduced in IL‐1β‐stimulated *Bmal1* ^−/−^ lung fibroblasts compared with IL‐1β‐stimulated *Bmal1* ^+/+^ lung fibroblasts, indicating that *Bmal1* supresses and induces a range of inflammatory mediators (Figure S2D). We further confirmed that the increased CXCL5 expression observed was specifically due to lack of *Bmal1* by performing siRNA knockdown in immortalized *Bmal1* ^+/+^ lung fibroblasts (Figure S2E–G). We achieved significant knockdown of *Bmal1* via siRNA in *Bmal1* ^+/+^ lung fibroblasts compared with the control siRNA (Figure S2E,F). Upon knockdown of *Bmal1*, *Cxcl5* mRNA expression significantly increased with IL‐1β stimulation in *Bmal1* ^+/+^ lung fibroblasts compared with corresponding control siRNA lung fibroblasts (*Bmal1* ^siRNA^; Figure S2G). We also investigated *Cxcl5* expression in *Cry1* ^−/−^ *Cry2* ^−/−^ immortalized lung fibroblasts and found that *Cry1* ^−/−^ *Cry2* ^−/−^ lung fibroblasts also display increased *Cxcl5* expression upon IL‐1β stimulation (Figure S2H). However, the fold change induction of *Cxcl5* mRNA expression was much greater with *Bmal1* deletion in lung fibroblasts compared with induction observed with loss of *Cry1/Cry2*. We observed significantly reduced expression of *Bmal1* in *Cry1* ^−/−^ *Cry2* ^−/−^ lung fibroblasts, therefore the increased *Cxcl5* mRNA expression may be attributed to the decreased expression of *Bmal1* in these cells (Figure S2I). Overall, these results confirmed the importance of *Bmal1* in controlling *Cxcl5* gene expression in lung fibroblasts.

To investigate if and to what extent the lung fibroblasts contribute to *Cxcl5* expression we observed in vivo (Figure 1D,E), we next carried out a serum shock on immortalized *Bmal1* ^+/+^ lung fibroblasts to synchronize the molecular clocks in vitro and analyze time‐of‐day responses (Figure S2J). Synchronized *Bmal1* ^+/+^ fibroblasts were stimulated with IL‐1β at 12 h‐post shock (which aligns to CT0) or 24 h‐post shock (which aligns to CT12), mirroring the CT times at which the mice were administered LPS intranasally (Figure 1A). Synchronized *Bmal1* ^+/+^ fibroblasts had significantly elevated CXCL5 protein production at CT12 compared with CT0 in vitro, consistent with our in vivo observations (Figure 2F). Although mRNA expression of *Cxcl5* was slightly more induced at CT12, there was no significant difference between *Cxcl5* expression at CT12 compared with CT0 (Figure 2E). Our findings infer a significant role of lung fibroblasts in mediating the time‐of‐day CXCL5 responses in the lung.

To further investigate the effect of *Bmal1* on the fibroblast immune response, we generated *Bmal1* ^*KD*^ mice using the tamoxifen‐*cre* model (Figure S2K). Postnatal suppression of *Bmal1* has advantages for our purposes over prenatal deletion, as embryonic knockout models of *Bmal1* have some developmental complications unrelated to the role of the transcription factor in the molecular clock.^37^ We dissected lungs from tamoxifen‐treated *Bmal1* ^*fl*/*fl*^ *ERcre* ^+^ and *cre* ^−^ *Bmal1* ^*fl*/*fl*^ control mice to obtain *Bmal1* ^*KD*^ and *Bmal1* ^*WT*^ primary lung fibroblasts respectively. There was a significant reduction of *Bmal1* mRNA in lung fibroblasts isolated from *Bmal1* ^*fl*/*fl*^ *ERcre* ^+^ mice compared with *cre* ^−^ *Bmal1* ^*fl*/*fl*^ control mice (Figure 2G). *Bmal1* ^*KD*^ and *Bmal1* ^*WT*^ primary lung fibroblasts were then stimulated with IL‐1β for 24‐h and assessed for *Cxcl5* expression via qPCR and ELISA (Figure 2H,I). *Bmal1* ^*KD*^ fibroblasts had significantly increased *Cxcl5* mRNA expression upon IL‐1β stimulation with no significant increase observed in IL‐1β‐stimulated *Bmal1* ^*WT*^ fibroblasts (Figure 2H). CXCL5 protein expression was comparable between the two genotypes (Figure 2I). Collectively, these results indicate that loss of *Bmal1* leads to an exaggerated inflammatory response in lung fibroblasts, characterized by enhanced *Cxcl5* expression.

### 3.3 Fibroblasts lacking *Bmal1* have increased phosphorylation of p65

**FIGURE 3:**
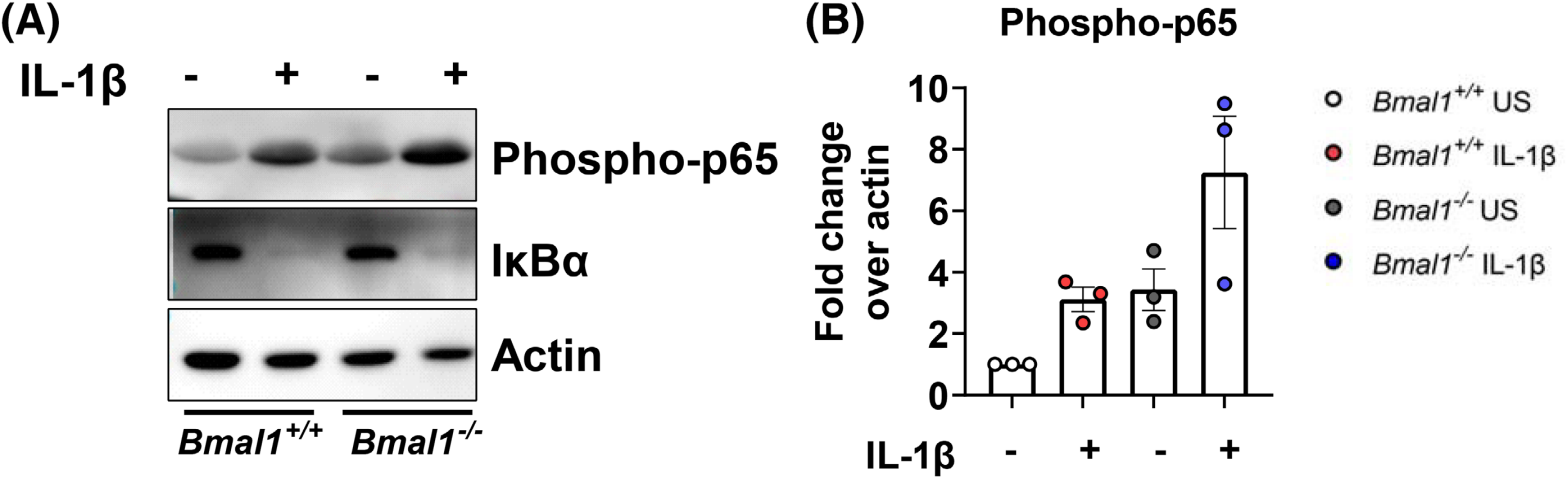
Immortalized lung fibroblasts lacking *Bmal1* have increased phosphorylation of the NF‐κB subunit p65. (A) Immunoblot and (B) densitometry for phospho‐p65 and IκBα in immortalized *Bmal1* ^+/+^ and *Bmal1* ^−/−^ lung fibroblasts left unstimulated or stimulated with IL‐1β (10 ng/ml) for 30 min (representative western blot shown; *n* = 3 separate experiments). Data are expressed as fold change over actin.

As increased expression of *Cxcl5* in IL‐1β‐stimulated *Bmal1* ^−/−^ lung fibroblasts was evident at the mRNA level, we next decided to investigate upstream signaling pathways regulating transcription factor activation. As previously discussed, the NF‐κB signaling pathway is necessary for *Cxcl5* transcription and its activity has been linked to circadian clock components.^38^, ^39^ Using a promoter analysis tool for detecting transcription factor binding motifs, we found enrichment of NF‐κB binding sequences in the *Cxcl5* promoter region (Figure S3A,B; Data Table S2), further validating the role of NF‐κB in *Cxcl5* transcription. We evaluated NF‐κB activation by stimulating immortalized *Bmal1* ^−/−^ and *Bmal1* ^+/+^ lung fibroblasts with IL‐1β for 30 min before analyzing phospho‐p65 and IκBα protein expression (Figure 3A,B). Interestingly, *Bmal1* ^−/−^ lung fibroblasts had elevated phosphorylation of phospho‐p65 both basally and with IL‐1β stimulation compared with *Bmal1* ^+/+^ fibroblasts, indicating that NF‐κB activity is increased in fibroblasts lacking *Bmal1* (Figure 3A,B).

### 3.4 Inhibition of NF‐κB signaling in lung fibroblasts decreases CXCL5 expression and neutrophil recruitment

**FIGURE 4:**
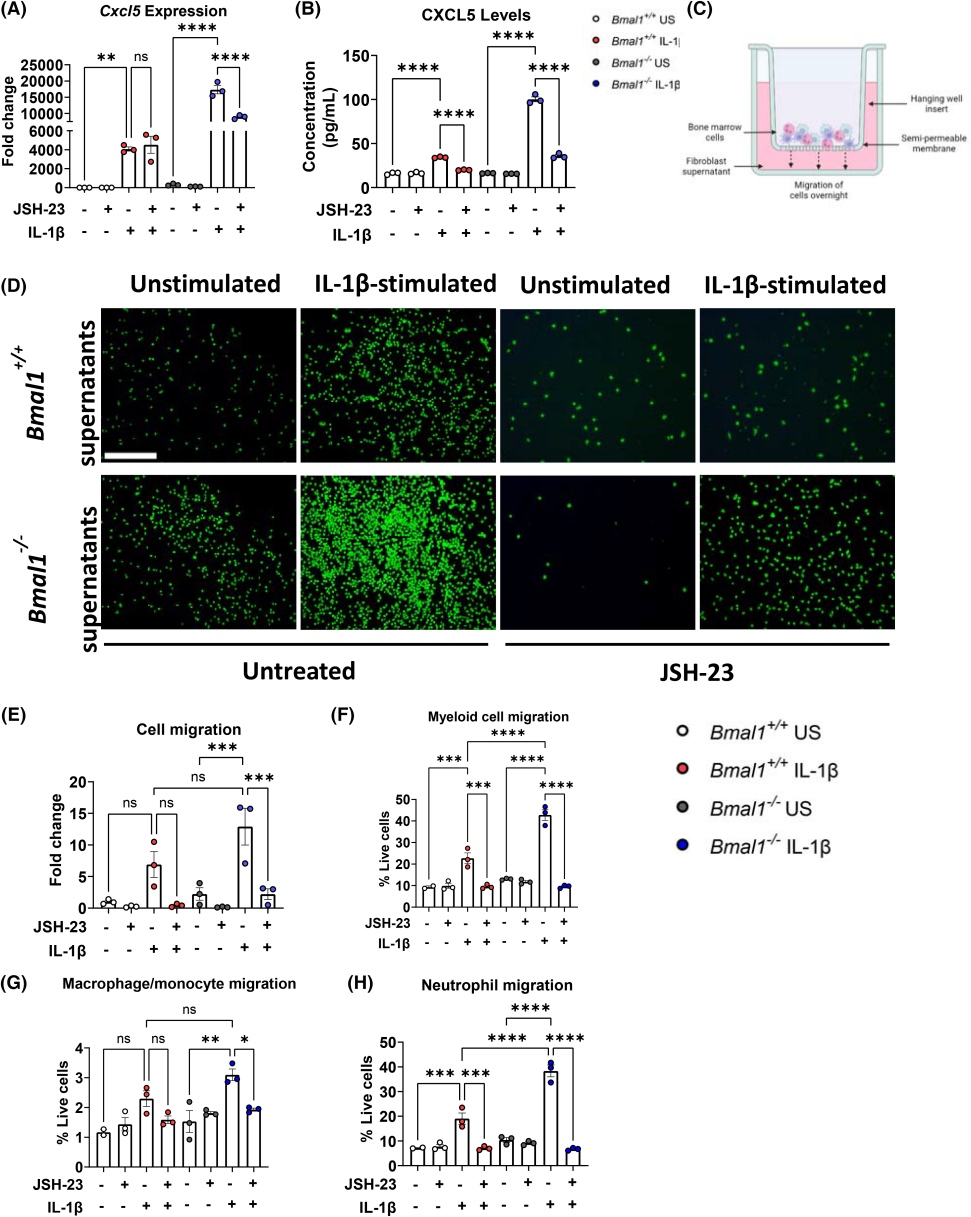
Inhibition of NF‐κB signaling in immortalized lung fibroblasts leads to decreased CXCL5 expression and decreased neutrophil recruitment. (A) mRNA and (B) protein expression of CXCL5 in immortalized *Bmal1* ^+/+^ and *Bmal1* ^−/−^ lung fibroblasts after vehicle or treatment with JSH‐23 (10 μM) for 1 h prior to being unstimulated or stimulated with IL‐1β (10 ng/ml) for 24 h (*n* = 3). (C) Schematic illustration of transwell migration assay. (D) Representative fluorescent microscopy images and (E) quantification of transwell migration assay of Calcein AM (2 μM) stained‐bone marrow‐derived cells after overnight migration of cells to conditioned media obtained from (A, B) (Data expressed as fold change over unstimulated/untreated *Bmal1* ^+/+^ samples; scale bar 200 μm). (F) Numbers of myeloid cells, (G) macrophages/monocytes, and (H) neutrophil migration from the same experimental set up as (D, E) quantified via flow cytometry (*n* = 3). Statistical analyses were conducted using one‐way analysis of variance (ANOVA) and Tukey's multiple comparisons test. Data are expressed as mean values per experimental group ± SEM. *p* < .05 (*), *p* < .01 (**), *p* < .001 (***), *p* < .0001 (****).

If increased NF‐κB activity was involved in modulating CXCL5 expression in IL‐1β‐stimulated *Bmal1* ^−/−^ lung fibroblasts, we reasoned that inhibition of NF‐κB activity should decrease CXCL5 expression in a *Bmal1*‐dependent manner. We inhibited NF‐κB activity using JSH‐23, an inhibitor which prevents p65 translocation to the nucleus and subsequent NF‐κB regulated transcription of *Cxcl5*.^40^, ^41^ JSH‐23 significantly decreased *Cxcl5* mRNA and protein expression in IL‐1β‐stimulated immortalized *Bmal1* ^−/−^ fibroblasts, and significantly decreased CXCL5 protein expression in IL‐1β‐stimulated *Bmal1* ^+/+^ fibroblasts (Figure 4A,B). We also examined the effect of JSH‐23 on CXCL1 mRNA and protein expression, another neutrophil recruiting chemokine that was elevated in IL‐1β‐stimulated immortalized *Bmal1* ^−/−^ fibroblasts (Figures S2D and S4C,D). Notably, NF‐κB inhibition had no effect on CXCL1 mRNA or protein expression in IL‐1β‐stimulated immortalized *Bmal1* ^+/+^ lung fibroblasts, while there was an increase in *Cxcl1* gene expression in corresponding immortalized *Bmal1* ^−/−^ lung fibroblasts that were not observed at the protein level (Figure S4C,D). This indicates that elevated NF‐κB activity in *Bmal1* ^−/−^ lung fibroblasts is driving the enhanced expression of CXCL5. The function of CXCL5 in vivo is to recruit immune cells, specifically neutrophils, to the site of infection. Thus, we decided to investigate immune cell recruitment to media harvested from immortalized *Bmal1* ^−/−^ and *Bmal1* ^+/+^ lung fibroblasts stimulated with or without IL‐1β (referred to as conditioned media; CM) using a transwell migration assay (Figure 4C). We stained migrated bone marrow‐derived cells with Calcein AM and quantified migration via fluorescent microscopy. We observed increased migration of cells to IL‐1β‐stimulated CM from *Bmal1* ^−/−^ lung fibroblasts compared with unstimulated samples, whereas no significant increase in migration to IL‐1β‐stimulated *Bmal1* ^+/+^ lung fibroblast CM was observed (Figure 4D,E). Use of JSH‐23 significantly reduced migration of bone marrow cells to IL‐1β‐stimulated *Bmal1* ^−/−^ lung fibroblast CM, inferring a key role for NF‐κB in lung fibroblast‐mediated CXCL5 recruitment of immune cells (Figure 4D,E). We next investigated specific innate immune cell recruitment to immortalized *Bmal1* ^−/−^ and *Bmal1* ^+/+^ lung fibroblast CM via flow cytometry (Figure S4A). Migration of myeloid cells was significantly elevated when IL‐1β‐stimulated *Bmal1* ^−/−^ lung fibroblast CM was used compared with corresponding CM from *Bmal1* ^+/+^ lung fibroblasts, whereas there was no significant increase in lymphocyte recruitment to either IL‐1β‐stimulated *Bmal1* ^+/+^ or *Bmal1* ^−/−^ fibroblast CM (Figures 4F and S4B). There was significantly increased migration of macrophages/monocytes to CM from *Bmal1* ^−/−^ lung fibroblasts stimulated with IL‐1β compared with control CM, whereas there was no significant migration to corresponding *Bmal1* ^+/+^ lung fibroblast CM compared with control CM (Figure 4G). Migration of neutrophils was significantly elevated when IL‐1β‐stimulated *Bmal1* ^−/−^ lung fibroblast CM was used compared with IL‐1β‐stimulated *Bmal1* ^+/+^ lung fibroblast CM (Figure 4H). While there was migration of both macrophages/monocytes and neutrophils to CM from IL‐1β‐stimulated *Bmal1* ^−/−^ lung fibroblasts, the percentage migration of neutrophils (mean 38.4%; Figure 4H) was much greater than that of migrating macrophages/monocytes (mean 3.1%; Figure 4G) indicating that CM from IL‐1β‐stimulated *Bmal1* ^−/−^ lung fibroblasts mainly attract neutrophils from the myeloid compartment. Inhibition of NF‐κB with JSH‐23 significantly reduced IL‐1β‐dependent neutrophil recruitment in both genotypes and was able to fully repress the elevated recruitment of cells observed with IL‐1β‐stimulated *Bmal1* ^−/−^ lung fibroblast CM to levels comparable with corresponding *Bmal1* ^+/+^ lung fibroblast CM with NF‐κB inhibition (Figure 4H). Collectively, this indicates that *Bmal1* inhibits NF‐κB‐driven *Cxcl5* expression in lung fibroblasts to impact on fibroblast‐mediated neutrophil recruitment during the inflammatory response.

## 4 DISCUSSION

**FIGURE 5:**
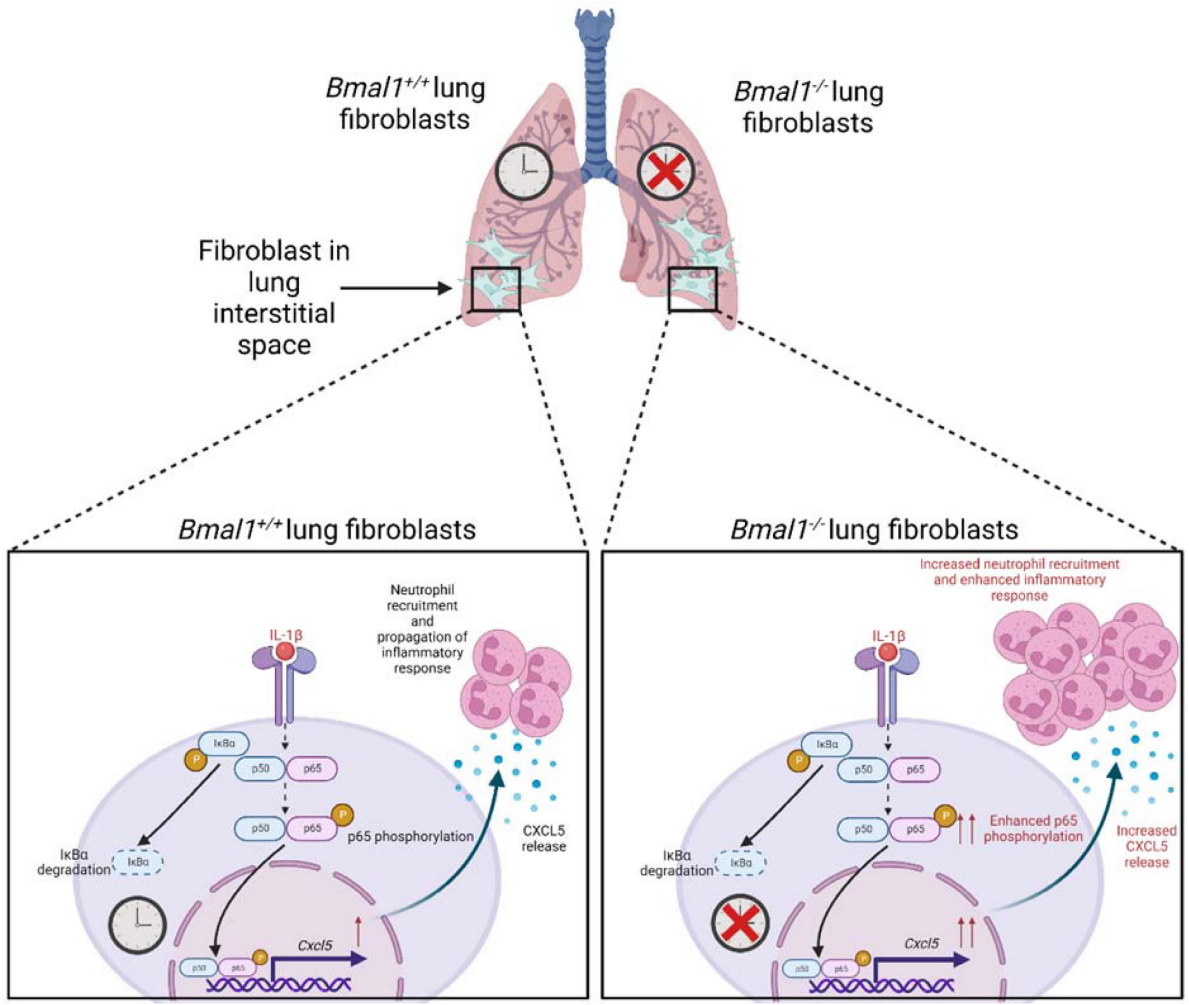
Schematic showing the findings outlined in this paper. Under inflammatory conditions, *Bmal1* ^−/−^ lung fibroblasts enhance phosphorylation of the NF‐κB subunit p65, leading to increased *Cxcl5* mRNA transcription. This subsequently leads to increased release of CXCL5, and heightened recruitment of neutrophils compared with *Bmal1* ^+/+^ lung fibroblasts. Created with BioRender.com.

Neutrophil‐mediated inflammation via airway epithelial cells has been well documented in chronic inflammatory diseases such as asthma and COPD.^31^, ^42^, ^43^ The main findings of our study are that the circadian clock in lung fibroblasts influences neutrophil recruitment via chemokine production, most likely via CXCL5, through *Bmal1*‐mediated suppression of NF‐κB activity. These findings are illustrated in Figure 5.

Circadian‐gated immune responses to the lung have been demonstrated in many models. Gibbs et al. demonstrated that deletion of *Bmal1* in airway epithelial cells leads to increased *Cxcl5* expression and increased neutrophil numbers in the BAL, and that rhythmic neutrophil recruitment is lost upon *Bmal1* deletion in these cells.^22^ However, numerous cell types exist in the lungs and airways which likely contribute to this temporal immune response. Fibroblasts are a prominent cell type found in the lung interstitium with important immunomodulatory roles and robust circadian rhythmicity.^44^, ^45^ However, how the lung fibroblast clock controls their immunomodulatory function is still unclear, and hence is the subject of our investigation. Differences exist between our study and that of Gibbs et al. Firstly, Gibbs et al. found neutrophil recruitment and CXCL5 expression was highest in the lung with LPS challenge at CT0 compared with CT12 in C57BL/6J, whereas we find significantly increased neutrophil recruitment and CXCL5 expression at CT12.^22^ There are experimental differences that exist between our studies. Firstly, Gibbs et al. used 2 mg/ml of aerosolized LPS, whereas we used 3 mg/ml of intranasal LPS.^22^ Secondly, the process of obtaining BAL can be a highly variable process that may account for differences observed in CXCL5 expression between these two studies. Finally, in this study we combined cell pellets from BAL into our digested lung suspension for neutrophil quantification, while Gibbs et al. looked at neutrophil recruitment solely in the BAL. Epithelial club cells in the pulmonary system have previously been found by this group to rhythmically produce CXCL5, contributing to increased CXCL5 in lung tissue at CT0.^22^ It is possible that CXCL5 production in the lung under basal conditions and at CT0 is controlled by club cells, as our study shows that lung fibroblasts do not produce high levels of CXCL5 without stimulation. Therefore, under steady state conditions in the lung, club cells may be the main drivers of neutrophil recruitment. However, our study indicates that under inflammatory conditions, lung fibroblasts are prominent CXCL5 producers and neutrophil recruiters, particularly at CT12. Although our results showed no significant difference in *Cxcl5* mRNA or protein expression in IL‐1β‐stimulated primary lung fibroblasts between CT0 and CT12, further investigations and a shorter stimulation (1 h as opposed to 24 h) of IL‐1β may be required to see statistically significant differences between *Bmal1* ^*WT*^ and *Bmal1* ^*KD*^ primary lung fibroblasts. Similar to our study, Sengupta et al. showed that the immune response is heightened in C57BL/6J mice intranasally infected with influenza virus at CT11 compared with CT23, and the CT11‐infected mice displayed increased mortality. Moreover, loss of *Bmal1* postnatally abolished the temporal survival and immune response.^15^ These studies demonstrate that loss of *Bmal1* leads to overactivation of inflammatory pathways within the lung.^15^, ^22^

The lung fibroblast clock has been shown to impact lung conditions such as pulmonary fibrosis. In mouse fibrotic lungs, circadian rhythms are amplified but asynchronous in comparison with non‐fibrotic tissue, and knockout of *Bmal1* in the pericyte (fibroblast) lineage reduced the amplitude in fibrotic tissue, while *Bmal1* deletion in club cells and macrophages had no effect.^6^ Moreover, deletion of *Nr1d1*, another core clock gene, specifically in lung fibroblasts increased the fibrotic response.^6^ In an arthritis model, *Bmal1* deletion in fibroblast‐like synoviocytes (FLS) led to increased *Cxcl5* expression and neutrophil recruitment into the joints of mice; however, the molecular mechanism that links *Bmal1*, *Cxcl5*, and neutrophil recruitment was not determined.^46^ Interestingly, loss of *Cry1* and *Cry2*, transcriptional repressors within the core molecular clock, in immortalized fibroblasts increased IL‐6 and TNF‐α expression which was dependent on constitutive NF‐κB activation through increased p65 phosphorylation.^47^ Here, we observed decreased expression of *Bmal1* in *Cry1* ^−/−^ *Cry2* ^−/−^ lung fibroblasts (Figure S2I). Our observed increase in p65 phosphorylation, *Cxcl5* expression, and neutrophil recruitment is not due to loss of *Cry1* and *Cry2*, as we show *Bmal1* ^−/−^ immortalized lung fibroblasts display increased *Cry1* and *Cry2* expression (Figure S2B). Taken together, these studies emphasize the importance of the fibroblast molecular clock in inflammation and lung disease, and the need to understand the downstream mechanisms associated with loss of each specific core clock gene.

There are several possible mechanisms by which *Bmal1* can impact on NF‐κB activity. NF‐κB activity has been shown to be circadian and peaks at Zeitgeber Time ZT6 (ZT0 being the start of lights‐on or the beginning of the rest phase) in mice administered the TLR5 agonist CBLB502.^39^ CLOCK has previously been shown to positively regulate NF‐κB transcriptional activity, with overexpression of CLOCK correlating with increased phosphorylation of p65. Spengler et al. hypothesize that BMAL1 may sequester CLOCK as a heterodimer in the cytoplasm, but when *Bmal1* expression is low or absent, CLOCK is free to interact with NF‐κB in the nucleus.^39^ Without *Bmal1*, CLOCK is free to enhance NF‐κB activity unhindered. Therefore, the mechanism of clock control of NF‐κB may rely on both BMAL1 and CLOCK. NF‐κB itself has been shown to modulate core clock genes, repressing BMAL1/CLOCK transcriptional activity in 293 T cells,^38^ while LPS has been shown to alter clock gene expression in the lungs by shortening the period length and enhancing recruitment of granulocytes such as neutrophils to the lungs in a circadian manner.^48^ Therefore, the relationship between inflammation, NF‐κB activity, and *Bmal1* are intrinsically linked.

The lungs are constantly being exposed to pathogens and neutrophilic inflammation is a common characteristic across many airway diseases such as COPD, asthma, and bacterial infection.^22^, ^43^, ^49^ Neutrophil recruitment is tightly regulated to mitigate tissue damage during an inflammatory response. Previous studies have shown that dampening of the pulmonary clock through smoking significantly reduces *Bmal1* gene expression in the lung, exacerbating lung inflammation,^14^ of which excessive neutrophil recruitment is a crucial feature.^24^ We found that lung fibroblasts are sensitive to circadian disruption, and consequently upregulate inflammatory pathway signaling to excessively recruit neutrophils. Lung fibroblasts may therefore be a potential target for treatments of lung diseases that have prominent neutrophilic inflammation and display circadian features.

In summary, we have found that lung fibroblasts are important regulators of neutrophil recruitment upon IL‐1β stimulation through CXCL5 production. This response is circadian gated and dependent on *Bmal1* regulation of NF‐κB activity. Our study links together several studies to further enhance our understanding of circadian immunology which may help unveil the therapeutics and interventions for chronic inflammatory lung disease.

## AUTHOR CONTRIBUTIONS

Shannon L. Cox conceived the study, designed and performed experiments, analyzed data, and wrote the manuscript; James R. O'Siorain performed experiments, assisted with analysis, and critically appraised the manuscript; Yan He, Ronan Lordan, Amruta Naik, and Soon Yew Tang assisted with in vivo experiments, Shaon Sengupta and Garret A. FitzGerald supervised the study and critically appraised the manuscript; Richard G. Carroll designed and supervised the study; Annie M. Curtis conceived and supervised the study and wrote the manuscript.

## DISCLOSURES

G.A.F. is the McNeil Professor of Translational Medicine and Therapeutics and a senior advisor to Calico Laboratories. The other authors declare no conflicts of interest.

## Supporting information


Dataset S1

Dataset S2

Figure S1
**Figure S2**.
**Figure S3**.
**Figure S4**.

## Data Availability

The raw data supporting the conclusions of this article will be made available by the authors, without undue reservation.
