# Supplementary material for: Circadian disruption in lung fibroblasts enhances NF‐κB activity to exacerbate neutrophil recruitment
Source: FASEB J. 2023 Jan 9;37(2):e22753. doi: 10.1096/fj.202201456R (PMC10107448; doi:10.1096/fj.202201456R)
Supplement: Supplementary file 3 — Figure S1 Figure S2. Figure S3. Figure S4. [file FSB2-37-0-s001.docx]

**Supplementary Figure 1
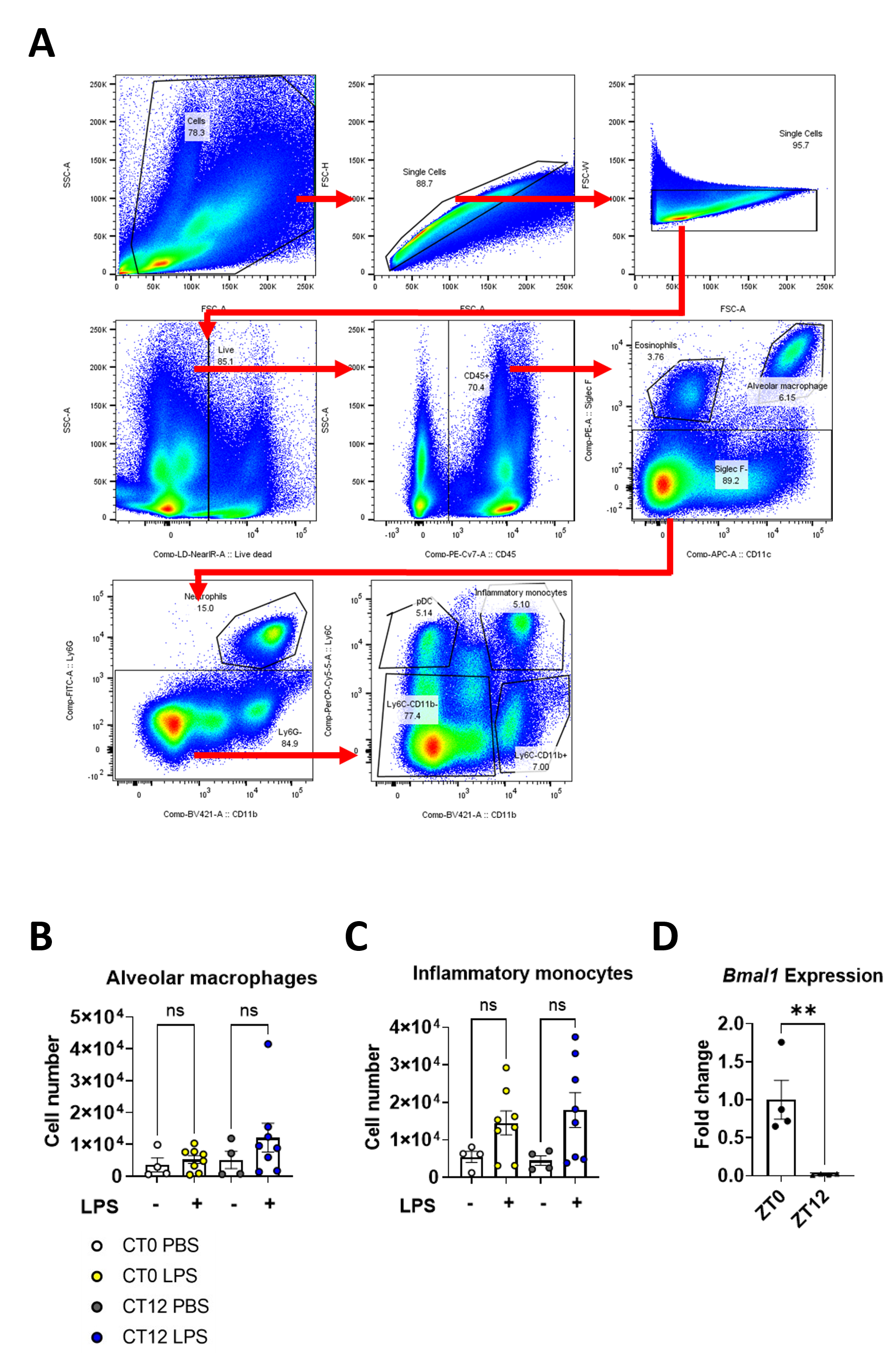
**

**Supplementary Figure 1**

(A) Gating strategy for lung neutrophils, alveolar macrophages, and inflammatory monocytes analysed via flow cytometry (Fig. 1F). (B) Alveolar macrophage and (C) inflammatory monocyte numbers recruited to the lung 24 hours after PBS (n=4) or LPS (3 mg/kg) (n=8) administration at CT0 or CT12. (D) *Bmal1* mRNA expression in murine lungs harvested at ZT0 or ZT12 (n=4). (B, C) Statistical analyses were conducted using one-way analysis of variance (ANOVA) with Tukey’s multiple comparisons test and (D) via unpaired *t* test. Data are expressed as mean values per experimental group ± SEM. p < 0.05 (*), p < 0.01 (**), p < 0.001 (***), p < 0.0001 (****).

**Supplementary Figure 2
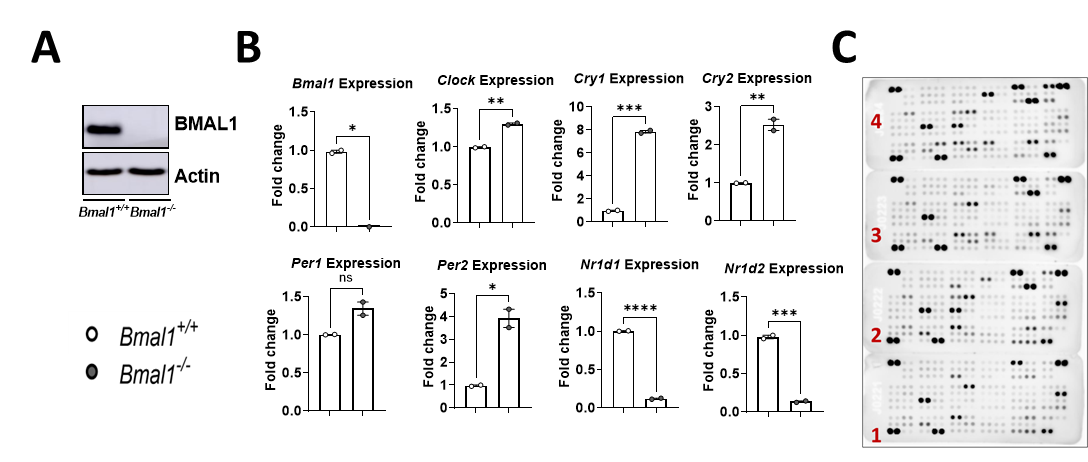
**

**
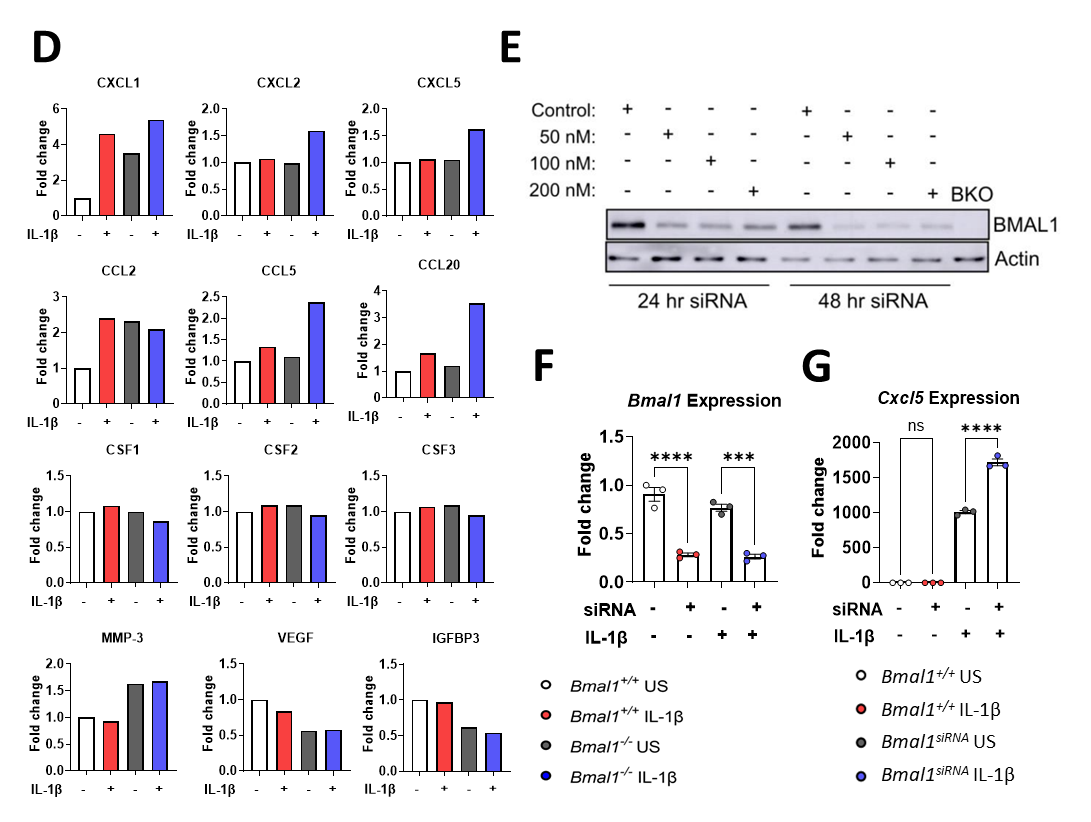
**

**
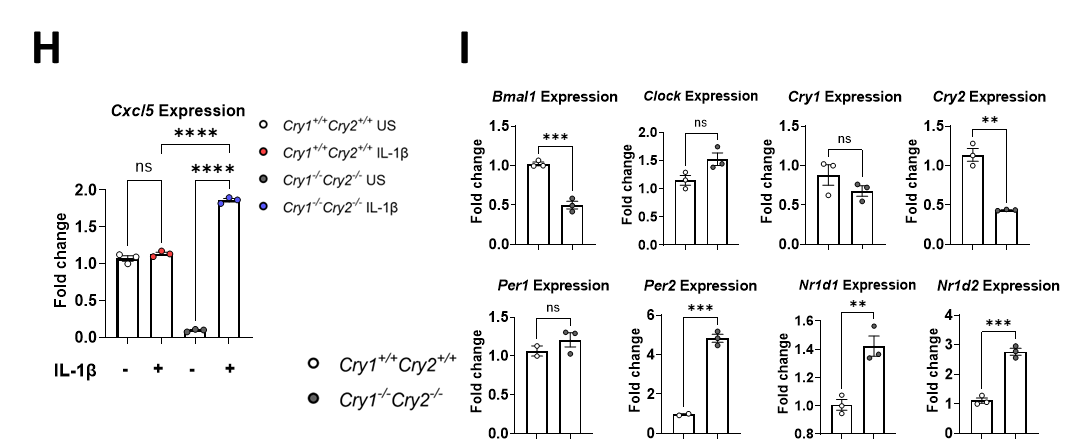
**

**
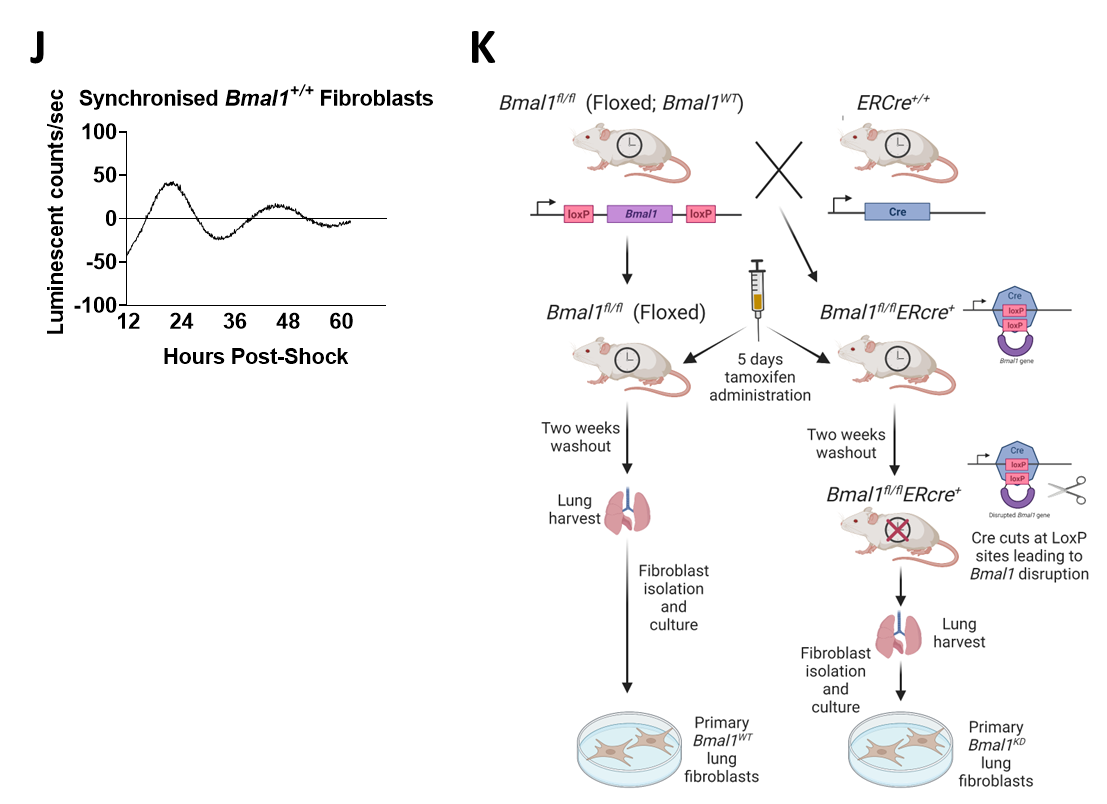
**

**Supplementary Figure 2**

(A) Immunoblot for BMAL1 expression in unsynchronised immortalised *Bmal1^+/+^* and *Bmal1*^-^*^/-^* lung fibroblasts. (B) mRNA expression of core clock genes in unsynchronised immortalised *Bmal1^+/+^* and *Bmal1^-/-^* lung fibroblasts analysed via qPCR (n=2). (C) Immunoblot of chemokine array from supernatants obtained from immortalised *Bmal1^+/+^* left (1) unstimulated or (2) stimulated with IL-1β (10 ng/mL), and *Bmal1^-/-^* lung fibroblasts left (3) unstimulated or (4) stimulated with IL-1β for 24 hours (Supplementary Data Table 1) (Fig. 2A, n=1). (D) Densitometry of most highly expressed chemokines/cytokines from chemokine array (Fig. 2B, n=1). (E) Immunoblot of BMAL1 expression in immortalised *Bmal1^+/+^* lung fibroblasts treated with *Bmal1* siRNA for indicated times and doses. Untreated *Bmal1^-/-^* lung fibroblasts loaded at end of immunoblot for control (n=1). (F) mRNA expression of *Bmal1* and (G) *Cxcl5* in immortalised *Bmal1^+/+^* lung fibroblasts treated with 50 nM *Arntl* siRNA for 24 hours prior to IL-1β stimulation or no stimulation for 24 hours (n=3). (H) mRNA expression of *Cxcl5* in immortalised *Cry1^+/+^Cry2^+/+^* and *Cry1^-/-^Cry2^-/-^* lung fibroblasts left unstimulated or stimulated with IL-1β for 24 hours (n=3). (I) mRNA expression of core clock genes in immortalised *Cry1^+/+^Cry2^+/+^* and *Cry1^-/-^Cry2^-/-^* lung fibroblasts (n=3). (J) Per2::luciferase immortalised *Bmal1^+/+^* lung fibroblasts were synchronised by serum shock and circadian rhythms were measured using LumiCycle technology (n=3). (K) Schematic illustrating generation of inducible *Bmal1^KD^* mice (*Bmal1^fl/fl^ERcre^+^*) and primary fibroblasts used in Fig. 2. (F-H) Statistical analyses were conducted using one-way analysis of variance (ANOVA) with Tukey’s multiple comparisons test and (B, I) via unpaired *t* test. Data are expressed as mean values per experimental group ± SEM. p < 0.05 (*), p < 0.01 (**), p < 0.001 (***), p < 0.0001 (****). (K) Created with BioRender.com.

**Supplementary Figure 3**


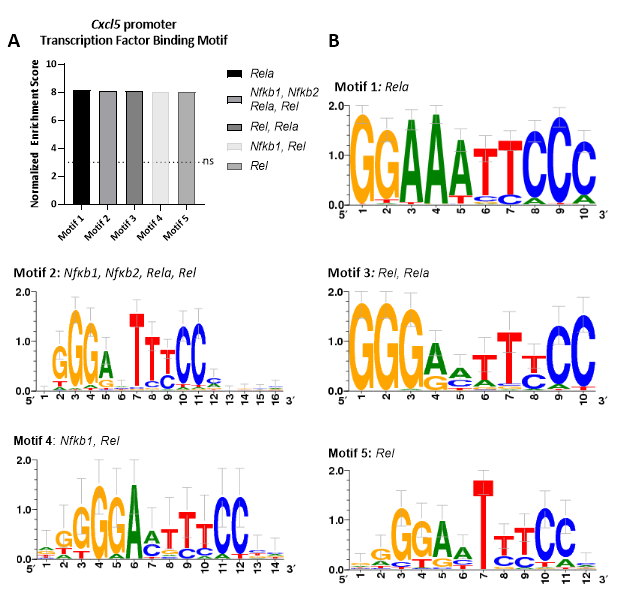


**Supplementary Figure 3**

(A) Transcription factor binding site enrichment for *Cxcl5* promoter region, 500bp proximal to the transcription start site. Selection of significantly enriched motifs based on the Normalized Enrichment Score (NES), which is calculated in RCIStarget for each motif based on the area under the curve distribution of all motifs within the gene promoter (X-mean/SD). (B) Motifs selected are representative of the top 5 motifs for which there are direct annotations for the transcription factors listed. Full list of enriched transcription factor binding site motifs see Supplementary Data Table 2.

**
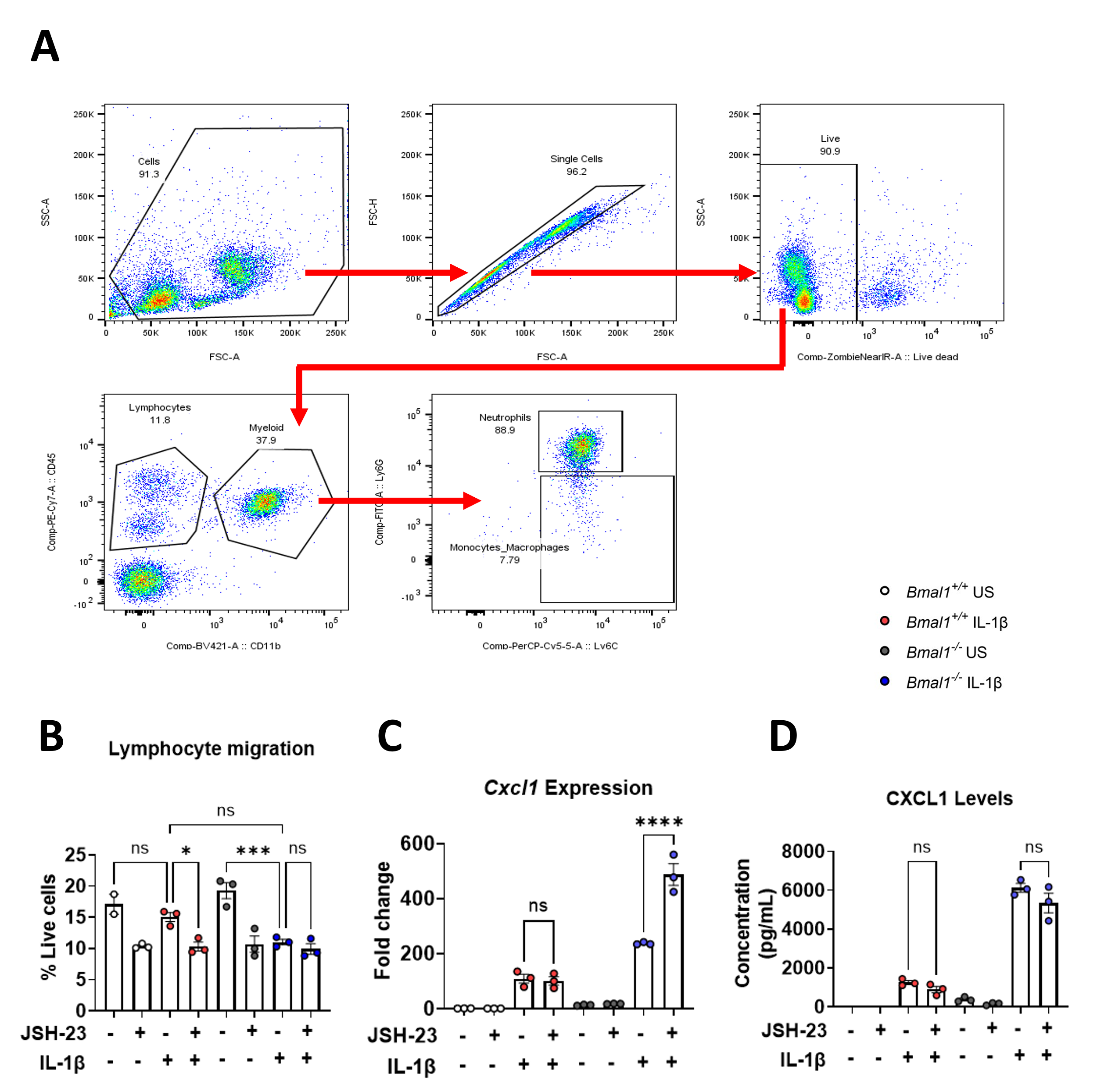
Supplementary Figure 4**

**Supplementary Figure 4**

(A) Gating strategy for transwell migration assay of migratory bone marrow-derived lymphocytes, myeloid cells, neutrophils, and monocytes/macrophages analysed via flow cytometry (Fig. 4F-H). (B) Lymphocyte migration in transwell migration assay analysed via flow cytometry (n=3). (C) mRNA and (B) protein expression of CXCL1 in immortalised *Bmal1^+/+^* and *Bmal1^-/-^* lung fibroblasts after vehicle or treatment with JSH-23 (10 µM) for 1 hour prior to being left unstimulated or stimulated with IL-1β (10 ng/mL) for 24 hours (n=3). Statistical analyses were conducted using one-way analysis of variance (ANOVA) with Tukey’s multiple comparisons test. Data are expressed as mean values per experimental group ± SEM. p < 0.05 (*), p < 0.01 (**), p < 0.001 (***), p < 0.0001 (****).
